# Nested case–control study of telomere length and lung cancer risk among heavy smokers in the β-Carotene and Retinol Efficacy Trial

**DOI:** 10.1038/s41416-018-0075-0

**Published:** 2018-04-19

**Authors:** Jennifer Anne Doherty, Laurie Grieshober, John R. Houck, Matt J. Barnett, Jean De Dieu Tapsoba, Mark D. Thornquist, Ching-Yun Wang, Gary E. Goodman, Chu Chen

**Affiliations:** 1 Huntsman Cancer Institute, University of Utah, Salt Lake City, UT 84112-5550 USA; 2 Division of Public Health Sciences, Fred Hutchinson Cancer Research Center, Seattle, WA 98109-1024 USA; 3 Department of Epidemiology, School of Public Health, University of Washington, Seattle, WA 98195 USA; 4 Department of Otolaryngology: Head and Neck Surgery, School of Medicine, University of Washington, Seattle, WA 98195 USA

**Keywords:** Risk factors, Cancer

## Abstract

**Background:**

Telomeres protect cells from genomic instability. We examined telomere length and lung cancer risk prospectively in heavy smokers.

**Methods:**

In a nested case–control study with 709 cases and 1313 controls, conditional logistic regression was used to evaluate associations between telomere length (global, chromosome 5p, and 13q) and lung cancer risk by histotype, controlling for detailed smoking history.

**Results:**

Risks of overall lung cancer and adenocarcinoma were suggestively elevated among individuals with telomere length in the longest tertile. No clear patterns were observed for other histotypes, or for chromosome 5p or 13q telomere length. Associations with adenocarcinoma were strongest among (OR, 95% CI for longest versus shortest tertile): former smokers (2.26, 1.03–4.96), individuals <65 years (2.22, 1.13–4.35), and women (2.21, 0.99–4.93).

**Conclusions:**

Our large study of heavy smokers adds additional evidence that long telomere length prior to diagnosis is associated with risk of lung adenocarcinoma, but not other histotypes.

## Introduction

Telomeres are chromatin structures that cap chromosomes, and critically short telomeres can cause chromosomal instability, enabling genetic changes in favor of carcinogenesis.^1^ At the same time, longer telomeres could result in enhanced proliferative potential, increasing the chance of accumulating mutations.^2,3^ Telomere length decreases with age, cigarette smoking,^4,5^ and oxidative stress and inflammation,^6–10^ and varies by chromosome arm.^11–13^ While three case–control studies suggested that short telomere length was associated with increased lung cancer risk,^14–16^ and a cohort study observed no association,^17^ a large case–control study^18^ and a pooled analysis of three nested case–control studies^19^ reported that longer telomere length is associated with increased risk, particularly for adenocarcinoma. Although the majority (80–90%) of lung cancers develop in current or former cigarette smokers,^20^ most smokers do not develop lung cancer. We evaluated whether telomere length (overall, and chromosome arms 5p and 13q), measured prior to diagnosis, was associated with lung cancer risk in heavy smokers, and if associations varied by histotype and other factors.

## Materials and methods

### Study population

The β-Carotene and Retinol Efficacy Trial (CARET) was a randomised, double-blinded, placebo-controlled chemoprevention trial of β-carotene and retinyl palmitate among 18,314 men and women at high risk of developing lung cancer.^21–23^ The intervention was stopped due to higher lung cancer incidence and overall mortality in the intervention arm after average follow-up of 4 years. Reports of cancer were confirmed through review of clinical records and pathology reports following a detailed protocol. The present study includes a subset of a previous nested case–control study designed using endpoint information collected during active follow-up (1985–2005).^24^ Briefly, participants who were free of lung cancer and provided a blood specimen (between 1994 and 1997) were eligible. Two lung cancer-free controls were matched to the 793 lung cancer cases on age (±4 years), sex, race/ethnicity, enrollment year (2-year intervals), baseline smoking status (current/former), occupational asbestos exposure, and length of follow-up. The current study includes the 717 cases and 1343 controls with sufficient DNA for telomere length assays. Institutional Review Boards for the CARET institutions approved study protocols, and all participants provided written informed consent.

### Laboratory methods

Blood was extracted using QIAamp DNA Blood Midi kits (Qiagen, Valencia, USA). Relative telomere length was measured using modified singleplex qPCR and normalisation per Aviv et al.,^25^ and the telomere to single-copy control gene ratio approach in McGrath et al.^26^ Individuals were measured in duplicate on two different runs. For differences >7%, samples were assayed again and the two closest values were averaged. Over 37 runs, the average coefficient of variation for the positive controls was 8.8%. Chromosome arm-specific 5p and13q telomere lengths were assayed adapting the modified STELA protocol of Xing et al.^27^ After quality control exclusions, the final analytic data set included 709 cases and 1313 controls. Detailed methods are included in Supplementary materials.

### Statistical analyses

Analyses were performed using SAS version 9.4 (SAS, Cary, NC). We examined associations between continuous global, 5p, and 13q telomere length and age, pack-years, cigarettes/day at blood draw, and body mass index (BMI, kg/m^2^) using Spearman correlations. Conditional logistic regression was used to examine associations between increasing tertiles of log2-transformed telomere length (based on the distribution in controls) and lung cancer risk, and by adenocarcinoma, squamous cell carcinoma, and small cell lung cancer. Odds ratios (ORs) and 95% confidence intervals (CIs) were calculated conditioning on the matching factors and adjusted for age, smoking status and pack-years at blood draw, and intervention arm. Associations were tested for linear trend. Stratified analyses of age, smoking status, sex, study arm, and time between blood draw and lung cancer diagnosis were performed. All statistical tests were two sided.

## Results

Baseline characteristics of this nested case–control study have been reported previously.^24^ Compared to other histotypes, a higher proportion of adenocarcinoma cases and controls were female (40% and 42%, respectively), and a lower proportion were current smokers at blood draw (57% and 55%, respectively) (Table 1). As expected, global and 5p telomere length were inversely associated with age at blood draw (Spearman correlations: −0.087, *p* = 0.00009, and −0.063, *p* = 0.006, respectively), but 13q telomere length was not. There was no association between global, 5p, or 13q telomere length and pack-years or cigarettes/day, perhaps due to the limited range of exposure. On average, blood was collected 5 years prior to diagnosis.

**Table 1:** Characteristics of lung cancer cases and controls, overall and by histologic type

	All^a^		Adenocarcinoma		Squamous cell		Small cell	
	Controls	Cases	Controls	Cases	Controls	Cases	Controls	Cases
	(*n* = 1313)	(*n* = 709)	(*n* = 338)	(*n* = 179)	(*n* = 264)	(*n* = 143)	(*n* = 224)	(*n* = 117)
*Matching variables*								
Age at baseline, mean years (SD)	59.3 (5.5)	60.3 (5.4)	59.1 (5.4)	60.1 (5.5)	59.7 (5.4)	61.1 (5.2)	59.6 (5.6)	60.4 (5.6)
45–54, *n* (%)	348 (27)	139 (20)	84 (25)	37 (21)	64 (24)	22 (15)	62 (28)	22 (19)
55–59, *n* (%)	342 (26)	184 (26)	98 (29)	51 (28)	67 (25)	36 (25)	48 (21)	30 (26)
60–64, *n* (%)	393 (30)	225 (32)	99 (29)	54 (30)	76 (29)	45 (31)	71 (32)	37 (32)
65–74, *n* (%)	230 (18)	161 (23)	57 (17)	37 (21)	57 (22)	40 (28)	43 (19)	28 (24)
Race, *n* (%)								
White	1247 (95)	669 (94)	327 (97)	172 (96)	249 (94)	134 (94)	215 (96)	112 (96)
Black	34 (3)	21 (3)	2 (1)	1 (1)	8 (3)	5 (3)	0 (0)	0 (0)
Other	32 (2)	19 (3)	9 (3)	6 (3)	7 (3)	4 (3)	9 (4)	5 (4)
Randomisation year, *n* (%)								
1985–1986	75 (6)	36 (5)	29 (9)	13 (7)	12 (5)	6 (4)	10 (4)	4 (3)
1987–1988	54 (4)	31 (4)	12 (4)	7 (4)	11 (4)	5 (3)	15 (7)	7 (6)
1989–1990	297 (23)	166 (23)	84 (25)	45 (25)	55 (21)	33 (23)	54 (24)	28 (24)
1991–1992	594 (45)	318 (45)	137 (41)	72 (40)	134 (51)	73 (51)	111 (50)	58 (50)
1993–1994	293 (22)	158 (22)	76 (22)	42 (23)	52 (20)	26 (18)	34 (15)	20 (17)
Asbestos exposure, *n* (%)	201 (15)	116 (16)	59 (17)	30 (17)	49 (19)	30 (21)	31 (14)	16 (14)
Current smoker at baseline, *n* (%)	974 (74)	523 (74)	224 (66)	121 (68)	211 (80)	112 (78)	167 (75)	88 (75)
Sex, *n* (%) female	467 (36)	236 (33)	142 (42)	71 (40)	69 (26)	36 (25)	95 (42)	48 (41)
*Other baseline characteristics*								
Intervention arm, *n* (%) assigned to active	694 (53)	386 (54)	185 (55)	96 (54)	138 (52)	73 (51)	114 (51)	64 (55)
Pack-years at baseline, mean (SD)	48.7 (20.8)	54.9 (21.3)	48.0 (21.8)	53.6 (20.3)	49.7 (20.5)	59.6 (25.4)	49.1 (21.2)	54.8 (20.1)
Years since quit smoking, mean (SD)	4.8 (5.7)	4.4 (4.7)	4.9 (6.4)	4.3 (5.1)	4.4 (4.4)	4.5 (3.2)	5.2 (5.2)	4.0 (4.6)
*Characteristics at blood draw*								
Age at blood draw, mean years (SD)	63.4 (5.8)	64.3 (5.6)	63.4 (5.9)	64.1 (5.7)	63.8 (5.7)	65.0 (5.6)	63.9 (5.9)	64.5 (5.7)
Current smoker at blood draw, *n* (%)	805 (61)	462 (65)	185 (55)	102 (57)	171 (65)	101 (71)	132 (59)	74 (63)
Pack-years at blood draw, mean (SD)	51.3 (21.3)	58.0 (21.9)	50.4 (22.2)	56.3 (20.5)	52.4 (21.0)	62.8 (26.3)	51.9 (21.8)	58.0 (20.3)
BMI (kg/m^2^), mean (SD)	27.7 (5.2)	27.1 (4.8)	27.7 (5.4)	27.3 (4.7)	28.0 (5.3)	27.2 (5.1)	27.6 (5.6)	27.3 (5.0)
<18.5 (%)	22 (2)	7 (1)	6 (2)	2 (1)	5 (2)	2 (1)	6 (3)	1 (1)
18.5–24.9 (%)	366 (28)	230 (33)	97 (29)	56 (31)	66 (25)	44 (31)	61 (28)	34 (29)
25.0–29.9 (%)	549 (42)	300 (42)	137 (41)	73 (41)	112 (42)	60 (42)	94 (43)	56 (48)
≥30.0 (%)	367 (28)	169 (24)	97 (29)	48 (27)	81 (31)	36 (25)	60 (27)	25 (22)

*SD* standard deviation, *BMI* body mass index.

^a^The “All” category includes adenocarcinoma, squamous cell, and small cell, as well as cases for whom histotype was missing (*n* = 270) and their matched controls (*n* = 487)

Risks of lung cancer overall and adenocarcinoma were suggestively elevated among individuals with global telomere length in the longest tertile. No clear patterns were observed between telomere length and other histotypes. The strongest associations with adenocarcinoma were observed among former smokers, individuals ages <65 years, and women (ORs (95% CI) for longest versus shortest tertile, respectively: 2.26 (1.03–4.96); 2.22 (1.13–4.35); and 2.21 (0.99–4.93)). Associations were similar regardless of intervention arm and time between blood draw and diagnosis (Table 2). Chromosome 5p and 13q telomere length were not associated with adenocarcinoma risk (Supplementary Table 1).

**Table 2:** Telomere length and lung cancer risk by histotype among heavy smokers, overall, and stratified by age, smoking status, sex, intervention arm, and time between blood draw and diagnosis^a^

	TL tertiles	All^b^				Adenocarcinoma				Squamous cell				Small cell			
		*n*_case_	*n*_Cont_	OR	(95% CI)	*n*_case_	*n*_Cont_	OR	(95% CI)	*n*_case_	*n*_Cont_	OR	(95% CI)	*n*_case_	*n*_Cont_	OR	(95% CI)
Overall																	
	1 (shortest)	221	434	1.00	(Ref.)	54	111	1.00	(Ref.)	47	87	1.00	(Ref.)	44	70	1.00	(Ref.)
	2	255	446	1.17	(0.92–1.47)	57	108	1.19	(0.73–1.94)	56	92	1.23	(0.73–2.07)	38	82	0.76	(0.44–1.32)
	3 (longest)	233	433	1.21	(0.95–1.55)	68	119	1.45	(0.88–2.37)	40	85	0.96	(0.54–1.70)	35	72	0.92	(0.51–1.66)
	*P*-trend	0.12				0.14				0.98				0.76			
Age at blood draw																	
≤65 years	1 (shortest)	98	230	1.00	(Ref.)	23	62	1.00	(Ref.)	17	45	1.00	(Ref.)	20	32	1.00	(Ref.)
	2	132	221	1.43	(1.03–1.98)	30	57	1.59	(0.80–3.14)	27	39	2.09	(0.97–4.55)	20	45	0.67	(0.31–1.48)
	3 (longest)	142	258	1.43	(1.03–1.98)	48	73	2.22	(1.13–4.35)	20	39	1.54	(0.65–3.64)	18	42	0.66	(0.28–1.55)
	*P*-trend	0.04				0.02				0.24				0.34			
>65 years	1 (shortest)	123	204	1.00	(Ref.)	31	49	1.00	(Ref.)	30	42	1.00	(Ref.)	24	38	1.00	(Ref.)
	2	123	225	0.90	(0.65–1.25)	27	51	0.85	(0.41–1.73)	29	53	0.70	(0.35–1.39)	18	37	0.82	(0.38–1.78)
	3 (longest)	91	175	0.84	(0.60–1.19)	20	46	0.60	(0.28–1.30)	20	46	0.58	(0.28–1.20)	17	30	0.90	(0.41–2.00)
	*P*-trend	0.33				0.19				0.13				0.78			
Smoking status at blood draw																	
Former smoker	1 (shortest)	67	159	1.00	(Ref.)	21	53	1.00	(Ref.)	13	23	1.00	(Ref.)	15	25	1.00	(Ref.)
	2	84	154	1.17	(0.77–1.79)	24	52	1.12	(0.51–2.46)	16	26	1.15	(0.40–3.30)	11	28	0.55	(0.18–1.65)
	3 (longest)	96	142	1.82	(1.20–2.77)	32	47	2.26	(1.03–4.96)	13	22	1.82	(0.50–6.58)	17	32	0.92	(0.34–2.52)
	*P*-trend	0.005				0.04				0.39				0.95			
Current smoker	1 (shortest)	154	275	1.00	(Ref.)	33	58	1.00	(Ref.)	34	64	1.00	(Ref.)	29	45	1.00	(Ref.)
	2	171	292	1.17	(0.88–1.54)	33	56	1.31	(0.69–2.49)	40	66	1.32	(0.72–2.43)	27	54	0.87	(0.47–1.64)
	3 (longest)	137	291	0.96	(0.71–1.31)	36	72	1.10	(0.58–2.12)	27	63	0.86	(0.44–1.69)	18	40	0.86	(0.41–1.83)
	*P*-trend	0.85				0.81				0.75				0.67			
Sex																	
Women	1 (shortest)	63	157	1.00	(Ref.)	15	45	1.00	(Ref.)	8	22	1.00	(Ref.)	20	34	1.00	(Ref.)
	2	87	159	1.32	(0.89–1.96)	27	51	1.65	(0.76–3.57)	13	22	1.94	(0.62–6.07)	14	34	0.63	(0.27–1.51)
	3 (longest)	86	151	1.52	(1.01–2.29)	29	46	2.21	(0.99–4.93)	15	25	1.51	(0.53–4.29)	14	27	0.83	(0.33–2.11)
	*P*-trend	0.05				0.05				0.49				0.62			
Men	1 (shortest)	158	277	1.00	(Ref.)	39	66	1.00	(Ref.)	39	65	1.00	(Ref.)	24	36	1.00	(Ref.)
	2	168	287	1.09	(0.82–1.46)	30	57	0.92	(0.48–1.77)	43	70	1.09	(0.59–2.02)	24	48	0.79	(0.38–1.65)
	3 (longest)	147	282	1.07	(0.79–1.46)	39	73	1.07	(0.56–2.02)	25	60	0.81	(0.40–1.65)	21	45	0.91	(0.41–2.01)
	*P*-trend	0.64				0.83				0.64				0.83			
Intervention arm																	
Active	1 (shortest)	121	233	1.00	(Ref.)	28	54	1.00	(Ref.)	22	43	1.00	(Ref.)	28	38	1.00	(Ref.)
	2	141	254	1.11	(0.81–1.52)	30	56	1.06	(0.53–2.12)	31	50	1.38	(0.66–2.92)	18	45	0.55	(0.26–1.16)
	3 (longest)	124	231	1.24	(0.89–1.73)	38	69	1.31	(0.64–2.67)	20	45	0.97	(0.45–2.12)	18	34	0.77	(0.35–1.72)
	*P*-trend	0.21				0.45				0.94				0.47			
Placebo	1 (shortest)	100	201	1.00	(Ref.)	26	57	1.00	(Ref.)	25	44	1.00	(Ref.)	16	32	1.00	(Ref.)
	2	114	192	1.19	(0.84–1.69)	27	52	1.16	(0.57–2.38)	25	42	1.07	(0.50–2.27)	20	37	0.96	(0.42–2.20)
	3 (longest)	109	202	1.17	(0.81–1.68)	30	50	1.46	(0.74–2.92)	20	40	0.91	(0.39–2.14)	17	38	1.06	(0.43–2.61)
	*P*-trend	0.41				0.27				0.86				0.91			
Time between blood draw and lung cancer diagnosis																	
0–6 years	1 (shortest)	147	285	1.00	(Ref.)	42	82	1.00	(Ref.)	32	63	1.00	(Ref.)	31	46	1.00	(Ref.)
	2	157	274	1.19	(0.89–1.59)	38	74	1.11	(0.62–1.99)	40	67	1.36	(0.73–2.53)	25	58	0.61	(0.31–1.23)
	3 (longest)	140	265	1.30	(0.95–1.79)	48	89	1.34	(0.75–2.39)	31	59	1.36	(0.69–2.67)	24	48	0.93	(0.42–2.02)
	*P*-trend	0.10				0.32				0.33				0.76			
>6 years	1 (shortest)	74	149	1.00	(Ref.)	12	29	1.00	(Ref.)	15	24	1.00	(Ref.)	13	24	1.00	(Ref.)
	2	98	172	1.16	(0.78–1.72)	19	34	1.47	(0.57–3.81)	16	25	0.83	(0.28–2.44)	13	24	1.19	(0.46–3.09)
	3 (longest)	93	168	1.17	(0.78–1.74)	20	30	1.75	(0.65–4.76)	9	26	0.42	(0.13–1.43)	11	24	1.05	(0.41–2.71)
	*P*-trend	0.47				0.28				0.17				0.91			

*n*_case_ number of cases, *n*_cont_ number of controls, *OR* odds ratio, *CI* confidence interval, *TL* telomere length.

^a^Conditional logistic regression models based on the matching factors (baseline age and smoking status, sex, race/ethnicity, enrollment year, asbestos exposure, and follow-up time) and adjusted for age at blood draw, smoking status at blood draw, pack-year at blood draw and intervention arm. Any of these adjustment variables was not included when the analysis stratified on that variable.

^b^The “All” category includes adenocarcinoma, squamous cell, and small cell, as well as cases for whom histotype was missing (*n* = 270) and their matched controls (*n* = 487).

^c^Information on stage was missing for 204 cases and 365 controls

## Discussion

In the largest nested case–control study to date of telomere length and lung cancer risk among heavy smokers, and the only study to evaluate chromosome arm-specific telomere length, we observed that long telomere length measured on average 5 years prior to diagnosis is associated with increased risk of adenocarcinoma but not other histotypes, particularly among women, former smokers, and individuals <65 years. These findings are remarkably consistent with the MD Anderson Cancer Center case–control study,^18^ and the pooled analysis,^19^ including: (1) largely non-smoking women from the Shanghai Women’s Health Study (SWHS);^28^ (2) male heavy smokers from the Alpha-Tocopherol, Beta-Carotene Cancer (ATBC) Prevention Study;^5^ and (3) men and women from the Prostate, Lung, Colorectal, and Ovarian Screening Trial (PLCO). Both observed strongest associations with adenocarcinoma among women, and Sanchez-Espiridion et al.^18^ additionally observed strong associations among individuals ages <60 years, and smokers with <30 pack-years.

Though our study is larger than the combined studies in Seow et al.,^19^ we observed generally smaller associations, which could be due to several factors. First, CARET participants were extremely heavy smokers, with median pack-years of 52 for cases and 47 for controls. ATBC also included heavy smokers, with median pack-years of 40 for cases and 33 for controls. In contrast, SWHS included largely never-smokers, and in PLCO, median pack-years was dramatically higher for cases than controls (46 and 5.5, respectively). Although the pooled analysis adjusted for pack-years, residual confounding is possible. Second, while these studies matched cases and controls by age and sex, we additionally matched on current/former smoking status and other factors, possibly reducing residual confounding. Third, in the pooled study, telomere length was assayed using multiplex qPCR, whereas we used singleplex. The multiplex method assays the relative telomere length components simultaneously (rather than separately) which reduces variation in DNA quantity, and therefore it may be less prone to non-differential misclassification. In a meta-analysis of prospective studies of all cancer types, stronger associations between long telomere length and cancer risk were observed among studies that used multiplex qPCR.^29^ Finally, ATBC and SWHS extracted DNA using phenol chloroform and PLCO used magnetic bead extractions,^19^ whereas we used QIAamp kits, which have been reported to yield shorter telomere length measurements.^29–31^

Studies of genetic risk scores representing long telomere length also report associations with lung cancer. Two studies using OncoArray data observed an association with adenocarcinoma but not squamous cell carcinoma,^32,33^ and increased risks of both adenocarcinoma and squamous cell carcinoma were reported in a study of non-smoking women.^34^ In the single prospective study that did not observe an association between telomere length and lung cancer risk, a genetic risk score for long telomere length was particularly associated with lung cancer, compared to other cancer types.^17^ In Haycock et al.,^33^ of the 22 cancer types examined, lung adenocarcinoma was among the most strongly associated with genetically determined telomere length. These studies may reflect long-term telomere length, and are unlikely to suffer from reverse causation and unmeasured confounding.

Our study contributes to growing evidence linking long telomere length with increased lung adenocarcinoma risk. It is unlikely that this is due to changes caused by the disease, since associations are observed when measured prior to diagnosis, and similar associations are observed for genetic risk scores. Our study (and ATBC) shows that telomere length can differentiate individuals at higher risk even among heavy smokers. Therefore, telomere length and its genetic determinants should be considered in risk stratification models of lung cancer.

## Electronic supplementary material

Final supplementary methods

Supplementary Table
