# Supplementary material for: Nested case–control study of telomere length and lung cancer risk among heavy smokers in the β-Carotene and Retinol Efficacy Trial
Source: Br J Cancer. 2018 Apr 19;118(11):1513–7. doi: 10.1038/s41416-018-0075-0 (PMC5988820; doi:10.1038/s41416-018-0075-0)
Supplement: Supplementary file 1 — Final supplementary methods [file 41416_2018_75_MOESM1_ESM.docx]

**Supplementary Materials and Methods**

**Study population**

Participants in this study were drawn from a case-control study nested within the multicenter β-Carotene and Retinol Efficacy Trial (CARET)^1^. CARET was a randomized, double-blinded, placebo-controlled chemoprevention trial to assess safety and efficacy of daily supplementation with β-carotene and retinyl palmitate among individuals at high risk of developing lung cancer^2-4^. Men and women ages 50-69 years were eligible for the trial if they were current or former smokers (i.e., quit within six years prior to enrollment) with a cigarette smoking history of ≥20 pack-years (n=14,254). Additionally, men ages 45-69 years with occupational asbestos exposure who were current or former heavy smokers (i.e., quit within fifteen years) were included (n=4,060). Extensive information about smoking history and other risk factors was collected through annual questionnaires, and dietary data was collected with a food frequency questionnaire every other year. Whole blood was collected at participant visits in 1994- 1997. Due to higher lung cancer incidence and overall mortality rates in the intervention versus placebo arm, the intervention was stopped in 1996.

The present study includes a subset of a nested case-control study designed using endpoint information collected during active participant follow-up (1985 – 2005) as described previously^1^. Briefly, all participants who provided a blood specimen and were free of lung cancer at the time of blood collection were eligible. Two lung cancer-free controls were matched to each of the 793 lung cancer cases on age (±4 years), sex, race/ethnicity, enrollment year (two year intervals), baseline smoking status (current or former), history of occupational asbestos exposure, and length of follow-up. Eighteen controls were excluded due to low DNA yield, and three cases were excluded because their diagnoses were later learned to be benign or carcinoid lung tumors. An additional case and two controls were excluded after genotyping due to discordant sex information, resulting in 789 cases and 1,566 controls. For the current study, 38 matched sets in which a sample from the case and/or both matched controls was insufficient for telomere testing were excluded, leaving 751 cases and 1,441 controls available to assay telomere length. Two cases were later determined not to have lung cancer and were included as controls. On average, blood was drawn 5 years prior to diagnosis for cases. The Institutional Review Boards for each of the participating CARET institutions approved all study protocols, and all participants provided written informed consent.

**Laboratory Methods**

Telomere length assessment by qPCR

Telomere length measurement was performed per Aviv *et al*, 2011, modified from the original singleplex method of Cawthon, 2002. In brief, two qPCR reactions for telomere (T) and a single copy gene (S) of human beta globin (HBB) were assayed separately on a 7900 HT Fast Real-Time PCR System (Life Technologies, Foster City, USA). The primers for the telomere PCR were *tel1b* (5’-CGGTTTGTTTGGGTTTGGGTTTGGGTTTGGGTTTGGGTT-3’) final concentration 100 nM and *tel2b* (5’-GGCTTGCCTTACCCTTACCCTTACCCTTACCCTTACCCT-3’) final concentration 900 nM with reaction conditions as follows: initial denature at 98˚ C for 2 minutes followed by 30 cycles of denature at 96˚ C for 1 second and annealing at 54˚ C for 1 minute with data collection. A dissociation curve was performed to assess target specificity. The primers for the human beta globin were *hbg1* (5’-GCTTCTGACACAACTGTGTTCACTAGC-3’) final concentration 300 nM and *hbg2* (5’-CACCAACTTCATCCACGTTCACC-3’) final concentration 700 nM with reaction conditions as follows: initial denature at 98˚ C for 2 minutes followed by eight cycles of denature at 95˚ C for 15 seconds, annealing at 58˚ C for 1 second, and extension at 72˚ C for 20 seconds, and followed by 35 cycles of denature at 96˚ C for 1 second, annealing at 58˚ C for 1 second, extension of 72˚ C for 20 seconds, and hold at 83˚ C for 30 seconds with data collection followed by a final dissociation curve run. Both PCR reactions were run with primers, SsoAdvanced Universal SYBR Green Supermix (Bio-Rad, Hercules, USA), sterile water, and 15 ng DNA sample in a final 10 µl reaction volume. A standard curve was generated for each run using dilutions of HeLa cell DNA (New England Biolabs, Ipswich, USA) of 39, 19.5, 9.75, 4.875, 2.44, and 1.22 nanograms. In addition, positive control DNA samples from cell lines (K562 (Promega, Madison, USA), TCL1301 (Sigma-Aldrich, St. Louis, USA), HeLa, in-house blood derived genomic DNA samples, and no template controls were run. All standard curve points and control samples were run in quadruplicate with aliquots of each stored at -20˚ C and used only for one run to avoid freeze / thaw events. All standard curves for each run had R^2^≥0.99.

We used the T/S ratio analysis approach of McGrath *et al*, 2007 and the normalization approach of Aviv *et al*, 2011. To control for inter-assay variability for each run, the T/S ratio of the positive control DNA sample was divided by the average T/S ratio of that control sample from 37 runs to obtain a normalizing factor. This was done for all of the positive control samples and the average normalizing factor was used to adjust the sample DNA T/S ratio within that run. Samples were tested in duplicate in each run and the average Ct was used to calculate the T/S ratios. The T/S ratios were measured in two separate runs for each sample and on different assay plates and in different well positions to minimize possible PCR positional effects. The two separate adjusted T/S ratios of each sample had a variation cut-off limit of 7% CV. Samples in which the two adjusted T/S ratios were greater were assayed a third time and the two closest values were used for analysis.

Chromosome arm-specific telomere length assay for 5p, 13q, and 17p

*Assay – Ligation, purification, long PCR, real-time qPCR*

We also assayed 5p, 13q, and 17p chromosome arm-specific telomere length, because these regions contain *TERT, RB1,* and *p53*, respectively. Briefly, we chose chromosome arm 5p because the *TERT* gene is the last gene before the telomere of chromosome 5 and *TERT* has been reported to autoregulate itself by interacting with the 5p telomere^8^. Chromosome 13q was selected because it contains the cell cycle checkpoint gene *RB1*, which when abrogated may allow damaged cells to escape from senescence, and when combined with telomere maintenance, increased cellular proliferation^8^. We developed these chromosome-arm-specific telomere assays by adapting the modified STELA protocol of Xing *et al*, 2009. We designed primers for 5p and 13q and used their published primers for 17p^9^. Due to high homology in the subtelomere (telomere adjacent) region, we used Repeatmasker (http://www.repeatmasker.org/) and a genomic alignment tool (<http://www.genome.ucsc.edu>) to create target sequence-specific primers and confirmed specificity by sequencing the fragment. We designed primers for a two-step PCR that first amplifies the chromosome arm of interest from the subtelomere to the telomere end in a long PCR reaction, then secondary qPCRs that target regions unique to the subtelomere and the telomere repeat. Samples were run in duplicate, and those that had Cq values with standard deviation > 0.3 were repeated. If the repeat run also had a standard deviation > 0.3, an average was used. Final Cq data for the telomere and telomere adjacent runs were adjusted for plate to plate variation by using data from the positive controls run on every plate.

Samples were assayed following the method outlined in Xing, *et al*, 2009 with some modification. In the first step, 200 ng of extracted genomic DNA are ligated with a pool of 240 pmol of Telorette primers (40 pmol of each of six Telorette primers (see enclosed table for primer sequences). Samples are incubated with Telorette primers in a 9700 GeneAmp PCR System (Applied Biosystems) 65 ˚C for 5 min, 60 cycles of 65 ˚C down to 35˚ C for 1 min with 0.5 ˚C decreases in temperature at each cycle, and 35 ˚C incubation for 5 min. T4 Ligation reaction mix is added to each sample including 20 U of T4 Ligase and [1X] T4 Ligase Reaction Buffer (New England BioLabs), and 6 % of PEG 6000 (Fluka) in a final reaction volume of 25 µL. Reactions are incubated at 35 ˚C overnight followed by a 65 ˚C incubation for 20 min for heat inactivation. Samples are purified post-ligation using ZR-96 Genomic DNA Clean & Concentrator-5 (ZymoResearch) . For each chromosome arm (5p, 13q, and 17p), long PCR reactions were done in duplicate for each ligated sample. Long PCR reactions include 3.5 µL of ligated DNA, 10 pmol of Teltail primer, 10 pmol chromosome arm target primer (see enclosed table for primer sequences), 1 U of LongAmp Hot Start Taq DNA polymerase and 1 X of LongAmp Taq Reaction Buffer (New England BioLabs), 75 µM of each dNTP (New England BioLabs), in a final reaction volume of 10 µL. Samples are incubated in 9700 GeneAmp PCR System (Applied Biosystems) 93 ˚C for 3 min, 35 cycles of 93 ˚C for 15 sec, 60 ˚C for 30 sec, and 68 ˚C for 20 min, followed by 30 min incubation at 68 ˚C. Real time PCR to assay for telomere length and telomere-adjacent target sequence are assayed on a 7900 HT Fast Real-Time PCR System (Applied Biosystems). Duplicate long PCR reactions for each chromosome arm sequence for each sample are pooled together. Three µL of each sample are diluted in 250 µL of sterile water then sequentially diluted an additional two more times as 3 uL in 250 µL sterile water. Final diluted sample are assayed in duplicate for the telomere assay and the telomere adjacent target sequence in separate reactions in the same real time PCR plate. For the telomere assay, 3 ul of diluted long PCR product is amplified in a 7 ul reaction with 0.5 pmol of Tel1b primer, 4.5 pmol of Tel2b primer, and [1X] SsoAdvanced Universal SYBR Green Supermix (BioRad). For the telomere adjacent assay, 3 ul of diluted long PCR product is amplified in a 7 ul reaction with 2 pmol of forward and 2 pmol of reverse primer specific to each chromosome arm (see table for primer sequences) and [1X] SsoAdvanced Universal SYBR Green Supermix (BioRad). Real-time PCR cycles include 98 ˚C for 2 min, 35 cycles of 98 ˚C for 5 sec and 60 ˚C for 1 min, followed by dissociation curve of 98 ˚C for 15 sec, 60˚ C for 1 min, and 98 ˚C for 15 sec. For quality control purposes, ten cell line DNA, a no template control, and cell line DNA without ligation primers, were run on every plate and carried through from initial ligation to the final qPCR step. For every plate, positive and negative controls were re-configured in different wells to account for potential well position effects. All primers for each step were aliquoted into individual tubes from a bulk dilution and stored at -20˚C and used for one time use. Long PCR was performed for each region of interest using HeLa cell line DNA (New England Biolabs) in one bulk reaction. Six point standard curve dilutions were created and aliquoted into tubes from the bulk reactions and stored at -20˚C and used for one time use. For each run, new aliquots of each dilution were included to generate standard curves for both the telomere and TAS assay. All standard curve runs had R^2^ ≥ 0.99.

We observed a higher percentage of samples including cell line positive controls and in-house genomic samples with telomere adjacent qPCR Cq above 33 in the 17p assay. Overall, this assay had a larger variation in predicted length than 5p and 13q. Dr. Harold Riethman sought to sequence the subtelomere regions of all chromosome arms^10^. The 17p region was reported to have variation in subtelomere length with a short 17ptel allele and a longer 17ptel allele with incomplete sequencing data according to the 1/12/2012 subtelomere assembly that had been maintained by Dr. Riethman (available upon request). Because of the missing sequencing data, we were not able to determine whether the variation is due to true underlying biology or a problem with the assay, so these results are excluded.

Primers

| tel1b (qPCR) | CGGTTTGTTTGGGTTTGGGTTTGGGTTTGGGTTTGGGTT |
| --- | --- |
| tel2b (qPCR) | GGCTTGCCTTACCCTTACCCTTACCCTTACCCTTACCCT |
| TELTAIL (LongPCR) | TGCTCCGTGCATCTGGCATC |
| TELORETTE1 (Ligation) | TGCTCCGTGCATCTGGCATCCCCTAAC |
| TELORETTE2 (Ligation) | TGCTCCGTGCATCTGGCATCTAATCCCT |
| TELORETTE3 (Ligation) | TGCTCCGTGCATCTGGCATCCCTAACC |
| TELORETTE4 (Ligation) | TGCTCCGTGCATCTGGCATCCTAACCC |
| TELORETTE5 (Ligation) | TGCTCCGTGCATCTGGCATCAACCCTA |
| TELORETTE6 (Ligation) | TGCTCCGTGCATCTGGCATCACCCTAA |
| 5pBfwd (qPCR) | TTTGGGTGTTACGTGTGCAT |
| 5pBrev (qPCR) | AGGGTTCAGTGTGGAAAACG |
| 5pDrev (LongPCR) | CGCAACGACAGTGAAGAAAA |
| 17pTASfwd (qPCR) | GAATCCACGGATTGCTTTGTGTAC |
| 17pTASrev (qPCR) | CCTCAGCCTCTCAACCTGCTT |
| 17p6 (LongPCR) | GGCTGAACTATATAGCCTCTGC |
| 13qTASfwd11_24-(LongPCR and qPCR) | GAACTCAAATGCAGCATTCCTAATACAT |
| 13qTASrevB11_24 (qPCR) | ACCTGAACCCTAACCCTCCA |

Data Analysis

Real time qPCR data was analyzed using SDS 2.4 software (Applied Biosystems). Quality control steps included examining positive control, NTC, and standard curve data. Clipped data files were then exported into EXCEL and read into LinReg software^11^ (v.2015.3) to calculate Cq and PCR efficiency for the telomere and TAS runs. Samples that had Cq values with standard deviation greater than 0.3 in their duplicate runs were repeated. In cases in which the repeat run also had standard deviation greater than 0.3, an average of both runs was used as the final determined Cq value. Samples with Cq values identified as indeterminate or above 34 were assigned values of 36 for analysis. Final Cq data for the telomere and TAS runs was loaded into Factor qPCR software^12^ (v.2015.2) divided into individual plate runs to account for plate to plate variation using values from three of the positive controls that were run on every plate. Adjusted Cq values were used in the final data analysis. Cq values from the TAS run were subtracted from Cq values from the telomere run and normalized to the mean (note: outlier samples were not included in calculation of the mean) to generate a final reported value (2 ^–ΔΔCq^).

Quality control exclusions

Samples were excluded for the following reasons: 1. low DNA concentration (32 cases, 100 controls); 2. HBB values >3 interquartile range (IQR) away from the median (5 cases, 26 controls); and 3. global telomere length >3 IQR away from the median in controls (3 cases, 4 controls), leaving 709 cases and 1,313 controls in analyses of global telomere length. With these exclusions, some controls had to be re-matched to cases based on the original matching criteria, and the age and enrollment year criteria were relaxed in order to allow matches for 2 controls. For the chromosome 5p and 13q assays, additional cases and controls were excluded due to: 1. telomere adjacent sequence Cq values >30, or no result (52 and 28, respectively); and 2. telomere length >3 IQR away from the median in controls (131 and 148, respectively). Again, where necessary cases and controls were re-matched, relaxing the age and enrollment year criteria to allow matches for 6 controls in each analysis.

Relative telomere length distribution in controls

The Kolmogorov-Smirnov and Anderson-Darling goodness-of-fit test p-values for global, 5p, and 13q telomere length among controls after quality control exclusions (n=1,313, 1,257, and 1,269, respectively) were ≤0.00002, indicating that the distribution is not Gaussian in terms of skewedness and kurtosis. The data are skewed right (positive) based on the positive skewedness values >0.61 for global, 5p, and 13q telomere length. The median values are also less than the mean values for all three telomere length measures. We therefore chose to apply a log-transform to the telomere length data in order to obtain more Gaussian distributions as is routinely done in the literature^13, 14^. Both the original and the log2-transformed relative telomere length cutoffs for controls are provided below.

| **Relative telomere length (T/S) cutoffs among controls** | | | | | | |
| --- | --- | --- | --- | --- | --- | --- |
| **Percentile** | **Original distribution** | | | **Log2-transformed distribution** | | |
|  | **Global** | **Chr5p** | **Chr13q** | **Global** | **Chr5p** | **Chr13q** |
| **Tertiles** | | | | | | |
| 33 | 0.83 | 0.82 | 0.85 | -0.27 | -0.29 | -0.24 |
| 67 | 1.16 | 1.10 | 1.08 | 0.21 | 0.14 | 0.12 |
| **Deciles** | | | | | | |
| 10 | 0.58 | 0.62 | 0.67 | -0.78 | -0.70 | -0.58 |
| 20 | 0.69 | 0.71 | 0.76 | -0.54 | -0.49 | -0.40 |
| 30 | 0.80 | 0.80 | 0.83 | -0.33 | -0.33 | -0.28 |
| 40 | 0.89 | 0.87 | 0.90 | -0.17 | -0.20 | -0.15 |
| 50 | 0.99 | 0.95 | 0.96 | -0.01 | -0.08 | -0.05 |
| 60 | 1.08 | 1.04 | 1.03 | 0.12 | 0.05 | 0.05 |
| 70 | 1.19 | 1.14 | 1.11 | 0.25 | 0.19 | 0.14 |
| 80 | 1.32 | 1.29 | 1.22 | 0.40 | 0.37 | 0.29 |
| 90 | 1.54 | 1.54 | 1.38 | 0.63 | 0.62 | 0.46 |

**References**

1. Sakoda L, Loomis M, Doherty J, Julianto L, Barnett M, Neuhouser M*, et al.* Germ line variation in nucleotide excision repair genes and lung cancer risk in smokers. *Int J Mol Epidemiol Genet*. 2012; **3**(1): 1-17.

2. Omenn GS, Goodman GE, Thornquist MD, Balmes J, Cullen MR, Glass A*, et al.* Effects of a combination of beta carotene and vitamin A on lung cancer and cardiovascular disease. *N Engl J Med*. 1996; **334**(18): 1150-1155.

3. Omenn GS, Goodman GE, Thornquist MD, Balmes J, Cullen MR, Glass A*, et al.* Risk factors for lung cancer and for intervention effects in CARET, the Beta-Carotene and Retinol Efficacy Trial. *J Natl Cancer Inst*. 1996; **88**(21): 1550-1559.

4. Goodman GE, Thornquist MD, Balmes J, Cullen MR, Meyskens FL, Jr., Omenn GS*, et al.* The Beta-Carotene and Retinol Efficacy Trial: incidence of lung cancer and cardiovascular disease mortality during 6-year follow-up after stopping beta-carotene and retinol supplements. *J Natl Cancer Inst*. 2004; **96**(23): 1743-1750.

5. Aviv A, Hunt SC, Lin J, Cao X, Kimura M, Blackburn E. Impartial comparative analysis of measurement of leukocyte telomere length/DNA content by Southern blots and qPCR. *Nucleic Acids Res*. 2011; **39**(20): e134.

6. Cawthon RM. Telomere measurement by quantitative PCR. *Nucleic Acids Res*. 2002; **30**(10): e47.

7. McGrath M, Wong JY, Michaud D, Hunter DJ, De Vivo I. Telomere length, cigarette smoking, and bladder cancer risk in men and women. *Cancer Epidemiol Biomarkers Prev*. 2007; **16**(4): 815-819.

8. Shay JW. Role of Telomeres and Telomerase in Aging and Cancer. *Cancer Discov*. 2016; **6**(6): 584-593.

9. Xing J, Ajani JA, Chen M, Izzo J, Lin J, Chen Z*, et al.* Constitutive short telomere length of chromosome 17p and 12q but not 11q and 2p is associated with an increased risk for esophageal cancer. *Cancer Prev Res (Phila)*. 2009; **2**(5): 459-465.

10. Riethman H, Ambrosini A, Castaneda C, Finklestein J, Hu XL, Mudunuri U*, et al.* Mapping and initial analysis of human subtelomeric sequence assemblies. *Genome Res*. 2004; **14**(1): 18-28.

11. Ruijter JM, Ramakers C, Hoogaars WMH, Karlen Y, Bakker O, van den Hoff MJB*, et al.* Amplification efficiency: linking baseline and bias in the analysis of quantitative PCR data. *Nucleic Acids Res*. 2009; **37**(6): e45-e45.

12. Ruijter JM, Ruiz Villalba A, Hellemans J, Untergasser A, van den Hoff MJB. Removal of between-run variation in a multi-plate qPCR experiment. *Biomolecular Detection and Quantification*. 2015; **5**: 10-14.

13. Seow WJ, Cawthon RM, Purdue MP, Hu W, Gao YT, Huang WY*, et al.* Telomere length in white blood cell DNA and lung cancer: a pooled analysis of three prospective cohorts. *Cancer Res*. 2014; **74**(15): 4090-4098.

14. Lapham K, Kvale MN, Lin J, Connell S, Croen LA, Dispensa BP*, et al.* Automated Assay of Telomere Length Measurement and Informatics for 100,000 Subjects in the Genetic Epidemiology Research on Adult Health and Aging (GERA) Cohort. *Genetics*. 2015; **200**(4): 1061-1072.
